# Tailoring anaesthetic strategies for diabetes research: Acepromazine vs. medetomidine in Aachen minipigs

**DOI:** 10.1371/journal.pone.0316570

**Published:** 2025-01-30

**Authors:** Sabrina Soares, Elisabeth Wühl, Alexander Schlund, Tobias Schneider, Kyra Sohns, Roman Rukwied, Martin Schmelz, Bettina Kränzlin

**Affiliations:** 1 Department of Experimental Pain Research, MCTN Mannheim, Heidelberg University, Mannheim, Germany; 2 Medical Faculty Mannheim, Heidelberg University, Mannheim, Germany; Scuola Superiore Sant’Anna, ITALY

## Abstract

Pre-established anaesthetic protocols in animal models might unexpectedly interfere with the main outcome of scientific projects and therefore they need to account for the specific research goals. We aimed to optimize the anaesthetic protocol and animal handling strategies in a diabetes-related-study exemplifying how the anaesthetic approach must be adjusted for individual research targets. Aachen minipigs were used as a model to test long-lasting skin glucose sensors for diabetic human patients. A total of 6 animals participated in two or three rounds of experiments. Each round lasted 2 months, with a maximum of 2 rounds per year. In each round, animals were anaesthetised 4 times: for glucose sensors insertion, twice for glucagon stress tests (GST), and a last time for removal of sensors. Acepromazine (ACE) was compared to medetomidine (MED) in association with butorphanol (BUT) and Ketamine (KET) and 4 parameters were analysed to define the optimum anaesthetic protocol including: sedation level, anaesthesia duration, effects on blood glucose and safety. ACE-BUT demonstrated a weaker sedative effect but reduced overall experimental time, minimized anaesthetic risk and minimally interfered with the glucose metabolism. The improvement obtained by animal conditioning and handling strategies applied in this study were not objectively estimated, although the aversion behavior was completely abolished. Based on the analysed parameters, the use of acepromazine is proposed to be superior when Aachen Minipigs are used specifically as a model for diabetes-related studies, albeit the recommendations for the anaesthesia of minipigs suggest otherwise.

## Introduction

The concern with the 3R’s (refinement, reduction and replacement) and the limitations of some traditional biomedical animal models that do not sufficiently translate the complexity of human physiology, anatomy and disease progression [1, 2] has led to increasing use of swine in the last two decades of biomedical research. Additionally, when long-term and chronic studies are necessary, minipigs are preferable in comparison to other livestock breeds of swine as they allow for a facile handling and reduce housing-associated costs due to the lower weight at mature age [3, 4]. Diabetes is a world-wide health issue and to date many questions related to the pathophysiology of the disease remain open [5]. It is estimated that around 13% of global deaths are related to diabetes and the disease accounts for 12% of global health expenditure. It is expected that the prevalence of diabetes in adults will reach more than 10% worldwide until 2040 [6].

In anaesthesia of human diabetic patients, effects of hypoglycaemia due to fasting, the cortisol release due to surgical stress and the effect of anaesthetics on glucose metabolism and circulation have to be carefully considered in order to maintain tight glycaemic control [5, 7]. In both humans and dogs, acepromazine is described as a sedative that does not interfere with glucose levels [8, 9]. No research paper on the effects of phenothiazines in the minipig glucose metabolism was found, so we decided to test acepromazine in comparison with medetomidine, both administered in association with butorphanol and supplemented with ketamine. We were aware of the possible anti-insulin hyperglycemic effect of α2-agonists [10, 11], but assessed medetomidine initially as superior to benzodiazepines or azaperone in the maintenance of stable glucose levels [12–15]. Induction was performed with propofol (PROP), and animals were maintained under general anaesthesia with isoflurane (ISO). The anaesthetic protocols were compared in relation to sedation level, anaesthesia duration, effects on blood glucose and safety. In light of the EU directive of 2010/63/EU recommendations [14], we attempted to reduce the number of animals, shorten the anaesthetic time and overall duration of the project while increasing research outcomes for the specific study. To minimize stress-induced cortisol release due to animal manipulation we optimized animal handling in between and during premedication (pre-med) administration.

Even if the animals in this study were not diabetic, the influence of anaesthetics on glucose metabolism was highly undesirable. The study required frequent and accurate blood glucose measurements (BGM) and the performance of glucagon stress tests (GST). Among the effects of glucagon administration on glucose metabolism and physiology, we can cite glycogenolysis, gluconeogenesis and lipolysis, all ultimately resulting in elevated blood glucose [15]. The induction of a hyperglycaemic peak was designed to evaluate the performance of long-lasting glucose sensors for use in diabetic patients.

## Material and methods

### Animals

In total, six Aachen minipigs were purchased from Heinrichs Tierzucht GmbH (Heinsberg, Germany) with an age of 12 months and approx. 40–50 kg body weight. The Aachen minipig is a cross-breed of the Vietnamese Potbelly, the Schwäbisch Hällisch Landpig, the German Landrace and the Minnesota minipig. Overall, the breed is physiologically comparable to Göttingen minipigs [16] already widely used for biomedical research.

Animal experiments were performed in accordance with German animal welfare legislation and the 2010/63/EU Directive on the protection of animals for scientific purposes and approved by the ethical committee of Regierungspräsidium Karlsruhe, Germany. The procedure of glucose measurement and GST-induced hyperglycaemia were rated as a minor procedure with no or only transient pain according to the Gaynor and Muir Prevention Scoring System [17]. No pain-related behaviour, including altered posture, reduced appetite or food intake and physical activity, or withdrawal from manipulation of the wounded area, was observed in the minipigs during this study. One minipig showing signs of pneumonia was excluded for treatment. Both incomplete anaesthetic records and health status were used as criteria for exclusion during this study.

### Housing and husbandry

Animals arrived at Interfakultäre Biomedizinische Forschungseinrichtung (IBF) in Heidelberg two weeks prior to the first round of experiments and were conditioned to contact with researchers and animal caretakers. The minipigs were kept in groups of two animals identified by numbers in bays (ca. 6m^2^) with straw bedding under controlled climatic conditions: 22°C±2°C, 50–60% relative humidity, 12-12-hour light cycle. As enrichment, gnawing rods, chains and pellet balls were offered. The animals were fed twice a day (SAF 130M, Raiffeisen) and water was available ad libitum.

Interaction with the animals aimed at conditioning them to the measures of glucose and pre-med injections using positive reinforcement and gentle handling, thereby reducing stress-induced effects [18]. The conditioning was performed by quiet and calm approaches, using dried raisins and brushing, for distraction or positive reinforcement. By the beginning of the experiments, the animals were used to the researchers, allowing for manipulation. The glucose measurements and IM injections were randomly intercalated with petting approaches and positive reinforcement, therefore reducing their expectation and stress levels related to them. In the morning prior to anaesthesia, capillary baseline glucose measurement was performed if the animals were calm and allowed for it without resistance. If animals resisted needle puncture, glucose levels from previous days were used as baseline to avoid stress immediately before the anaesthetic procedure. The pre-medication was injected intramuscularly (IM) 1-3cm behind the ear into the lateral cervical muscle region while brushing and a few raisins were provided to distract the animals from the injection pain.

### Anaesthesia

The minipigs were submitted to 12 hours fasting without water withdrawal in accordance with the Society for Laboratory Animal Science (GV–SOLAS). The animals received medetomidine 10–25 μg/Kg (Sedator^®^, Dechra, Germany) or acepromazine 0.6–1.1 mg/Kg (Tranquisol^®^ P, cp-pharma, Switzerland) both combined with butorphanol 0.3–0.5 mg/Kg (Butorgesic^®^, cp-pharma, Switzerland) (MED-BUT or ACE-BUT, respectively). After pre-med the pigs were left for 10–15 minutes in a calm environment and the sedative effect was assessed by evaluation of posture and head position [19]. Animals standing and alert, with the head in a prone position were supplemented with ketamine 2.5–10 mg/kg (Ketamin^®^, Medistar, Germany) adjusting dosage based on temperament [20].

After sedation, the animals were moved into the surgical room using adapted stretchers and the ear vein accessed. Ringer Acetate 5 ml/Kg/h (Deltajonin^®^, Deltamedica, Germany) was administered throughout the whole procedure. Electrocardiogram (ECG) (Lohmeier M111, Lohmeier, Germany), exhaled carbon dioxide (EtCO2) (Dräger, Dräger Medical, Germany), oxygen saturation (SPO_2_) (Pulox^®^, Contec Medical Systems, China), non-invasive blood pressure (NIBP) (OMROM, OMROM Healthcare Europe, Holland), and rectal temperature (ama-digit, Precision, Germany) were monitored until full recovery from anaesthesia. Atropine sulfate (Atropinsulfat, B Braun, Germany) was administered intravenously at concentrations ranging from 0.01–0.02 mg/Kg when HR dropped under 60 b.p.m. The intubation was performed after inducing general anaesthesia with propofol (Narcofol^®^, cp-pharma, Switzerland) intravenously at a dosage of 0.5–3 mg/Kg. Lidocaine Gel 2% (Xylocain^®^, Gel 2%, Aspen, Germany) was applied to the tip of an endotracheal tube number 6.5–7.0 (ENDO-BREEZER, Servoprax, Germany) to attenuate intubation-induced airway responses. General anaesthesia was maintained with 0.5–1.5%vol of isoflurane in oxygen (1–2 L/min) (Isoflurane CP^®^, cp-pharma, Switzerland) in a rebreathing system. A volume-controlled ventilation mode was delivered with a E-Vent^®^ Plus (Dräger Medical, Germany); the tidal volume was defined as 10–15 ml/Kg volume of oxygen to 100–200 ml/Kg and the respiratory frequency was kept in a range between 8–20 rpm to maintain capnography within physiological range 40–50 mg/dL [21]. In two cases, Atipamezole (Revertor^®^, cp-pharma, Switzerland) was administered to revert the effect of Medetomidine and wean the animals from the ventilator [22]. No dysphoric effects, which may cause increased levels of stress and consequently cortisol, were observed during the recovery phase from anaesthesia.

### Study design

The diabetes-related study aimed to establish a comparison of glucose levels obtained by traditional glucometer measurements from a long-term skin sensor under test. The sensors were manufactured with a needle of approximately 1 cm inserted on the lateral abdominal skin. Each animal carried around 30 sensors, protected by plastic covers stitched on the skin and a jacket fabricated specifically for the minipigs. GSTs were performed under anaesthesia to compare the hyperglycemic peaks with baseline and assess the ability of the skin sensors to detect high glucose levels. Between 2–3 mg of glucagon (GlucaGen^®^, Novo Nordisk^®^, Germany) were injected intravenously to induce hyperglycemia.

**Fig 1:**
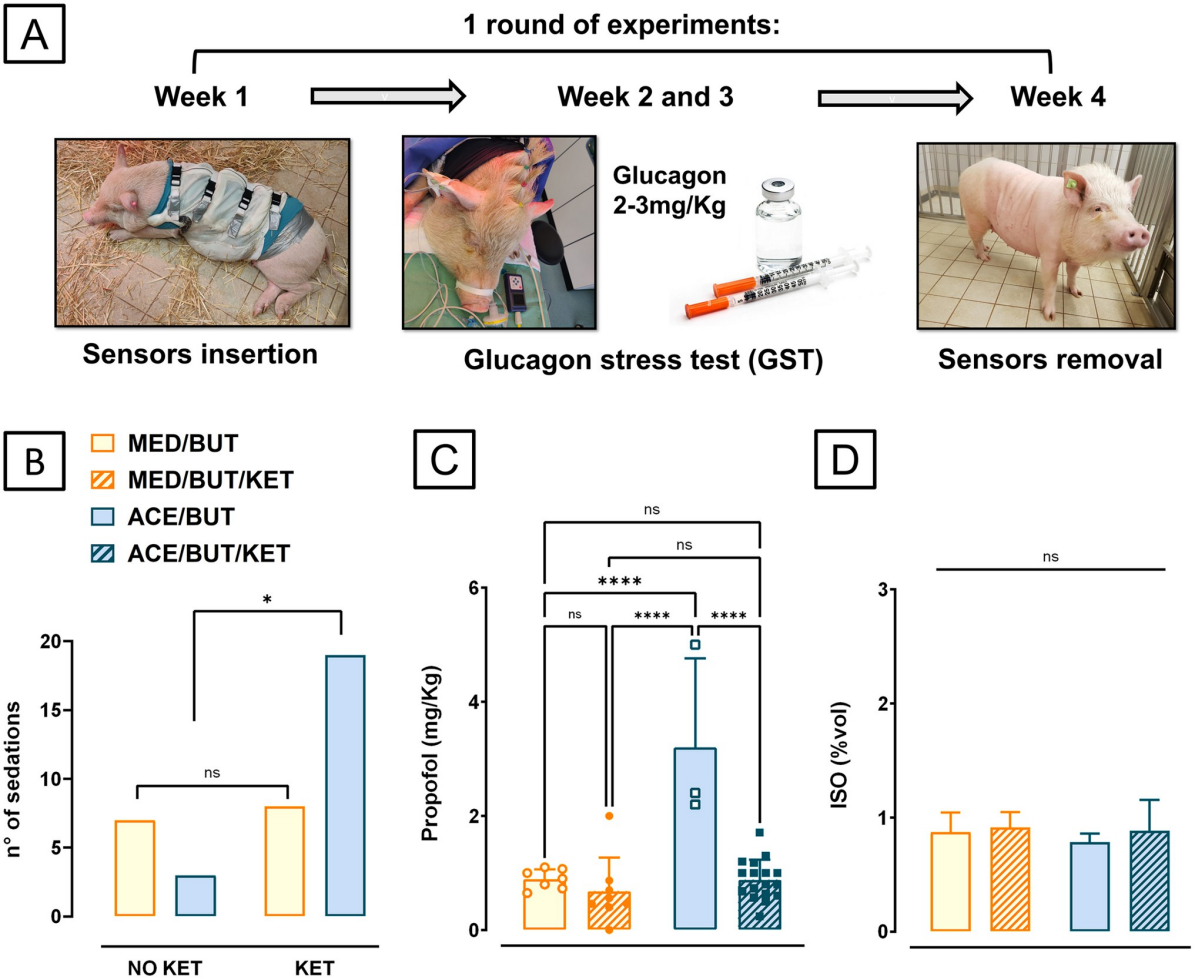
Pre-medication effect on sedation level. (A) The study was divided into rounds, each round comprising 4 anesthetic procedures: one for sensor insertion, two for GST and a last one for sensor removal. (B) The need to supplement the pre-med with Ketamine was significantly correlated with the usage of ACE-BUT, indicating a lower sedative capacity (Chi-squared t-test, *p = 0.*026)*. The dosage of propofol required to induce general anesthesia was higher when ACE-BUT (n = 3) was administered compared to MED-BUT (n = 7). (C) More propofol was also needed when comparing ACE-BUT to ACE-BUT-KET (n = 19) and to MED-BUT-KET (n = 8) (Repeated measures one-way ANOVA, Bonferroni post-hoc test, ****p<0.001). (D) The concentration of isoflurane in %vol for maintenance of general anesthesia was not affected by the pre-med choice (Repeated measures one-way ANOVA, Bonferroni post-hoc test, *p = 0*.*87*). Error bars represent standard deviations.

Glucose measurements were performed at an interval of 20 minutes with a glucometer (Accu-Check, Roche, Germany), starting before propofol administration and during the whole anaesthetic procedure, until animals were extubated. The GST tests could only begin when glucose was close to baseline, measured during the pre-anaesthetic period and in the days between procedures. Three consecutive blood glucose measures around baseline value in an interval of 20 minutes after the hyperglycemic peak drop were defined as a parameter for termination of anaesthesia and considered as a full return to basal condition. The glucose measurements obtained by the long-lasting sensors and the comparison with traditional needle punctures are the subject of another study. The project lasted around 2 years and was divided into 2 rounds of experiments per year with each round of experiments consisting in daily glucose measurements. Under general anaesthesia, 2 GST tests were performed at an interval of 2 weeks, and another 2 anaesthetic procedures were performed for the insertion and removal of the glucose sensors (Fig 1A). In order to maximize the read-out within the quite small group of 6 animals, each of them received approximately 30 long-lasting sensors distributed on both sides of thoraco-abdominal area. All animals underwent at least 2 rounds of experiments and either ACE-BUT or MED-BUT were randomly administered to the animals in a crossover design, making sure each animal received both pre-medication protocols during each round of assessment. Randomization was performed using the RAND function from MS Excel. The experimental anaesthetic protocols of ACE-BUT or MED-BUT were compared according to 1) The level of sedation: measured indirectly by the need to supplement the sedation with Ketamine in alert animals, the dosage of propofol needed to induce anaesthesia and the need of higher isoflurane concentration to maintain a desirable level of general anaesthesia. 2) The time to GST after general anaesthesia induction and the overall duration of the anaesthetic procedure, representing the overall success in the anaesthetic planning to deliver a shorter anaesthetic procedure. 3) The blood glucose measurements along the anaesthetic procedure as a measure of improved outcome. 4) The safety of both pre-meds: evaluated by the effect in reducing heart rate during the anaesthetic procedure, and therefore the need to administer anticholinergics to reestablish normal heart rate.

A total of 9 anesthetic procedures were excluded from the final analysis, due to lack in glucose measurements recording.

### Statistical analysis

Statistical analyses were performed by Prism 10.3.1 The data is normally distributed according to Shapiro-Wilk test (*p*>0.05). Bonferroni post-hoc tests were used in multifactorial repeated measures ANOVA and statistical significance was defined by a 95% confidence interval (p<0.05) when comparing sedation levels for the different pre-meds. Correlation among the pre-med and the need of supplementation with ketamine was performed by a Chi-squared t-test. The influence of the different pre-meds on anaesthetic time and the interference of atropine in glucose levels were evaluated by a Two tailed t-test, confidence interval of 95% (p<0.05). Data is presented as mean (SD). The sample size was calculated using G*Power software 3.1.9.4 for an effect size of 1 in a confidence interval of 0.95 with a power of 0.83. R software (version 4.2.2) with the RStudio GUI (version 2022.07.2 build 576) was used to calculate the effect size.

## Results

The rounds of experiments included two anaesthetic procedures without GST testing for sensor insertion and removal and two anaesthetic procedures with GST tests (Fig 1A), accompanied by daily capillary blood glucose measurements as mentioned previously. Both medetomidine “MED-BUT” and acepromazine “ACE-BUT” were compared, trying to establish an optimum protocol for our diabetic-related case.

A total of 15 anaesthetic procedures using MED-BUT and 22 using ACE-BUT were analysed, excluding incomplete monitoring records from the results. Ketamine was supplemented to the pre-med protocol to improve sedation when the animals were still alert and standing 15 minutes after sedation, and cannulation of the ear vein was not possible without a stressful approach. The need for supplementation with ketamine positively correlates with ACE-BUT (Chi-squared t-test, *p = 0*.*026*), meaning an increased need for supplementation when ACE-BUT was used (Fig 1B). Ketamine was supplemented in 19 out of 22 animals that received ACE-BUT and in 8 of the 15 animals from the MED-BUT group. The dosage of propofol used for inducing general anaesthesia was about 3-fold higher for ACE-BUT 3.2 (SD = 1.56) mg/Kg, compared to MED-BUT 0.89 (SD = 0.35) mg/Kg. More propofol was also required for ACE-BUT, compared to ACE-BUT-KET 0.88 (SD = 0.35) mg/Kg as well as MED-BUT-KET 0.68 (SD = 0.59) mg/Kg (Repeated measures one-way ANOVA, Bonferroni post-hoc test, *p*<0.001). There was no difference in the administered dosage of propofol to induce anaesthesia and intubate the animals, when comparing MED-BUT and MED-BUT-KET (Fig 1C). The concentration of isoflurane in %vol for maintenance was not affected using ACE-BUT nor MED-BUT, with or without supplementation with Ketamine. The %vol was 0.78 (SD = 0.08) for ACE-BUT 0.89 (SD = 0.27) for ACE-BUT-KET, 0.87 (SD = 0.17) for MED-BUT and 0.91 (SD = 0.13) for MED-BUT-KET (Repeated measures one-way ANOVA, Bonferroni post-hoc test, *p* = 0.87) (Fig 1D).

**Fig 2:**
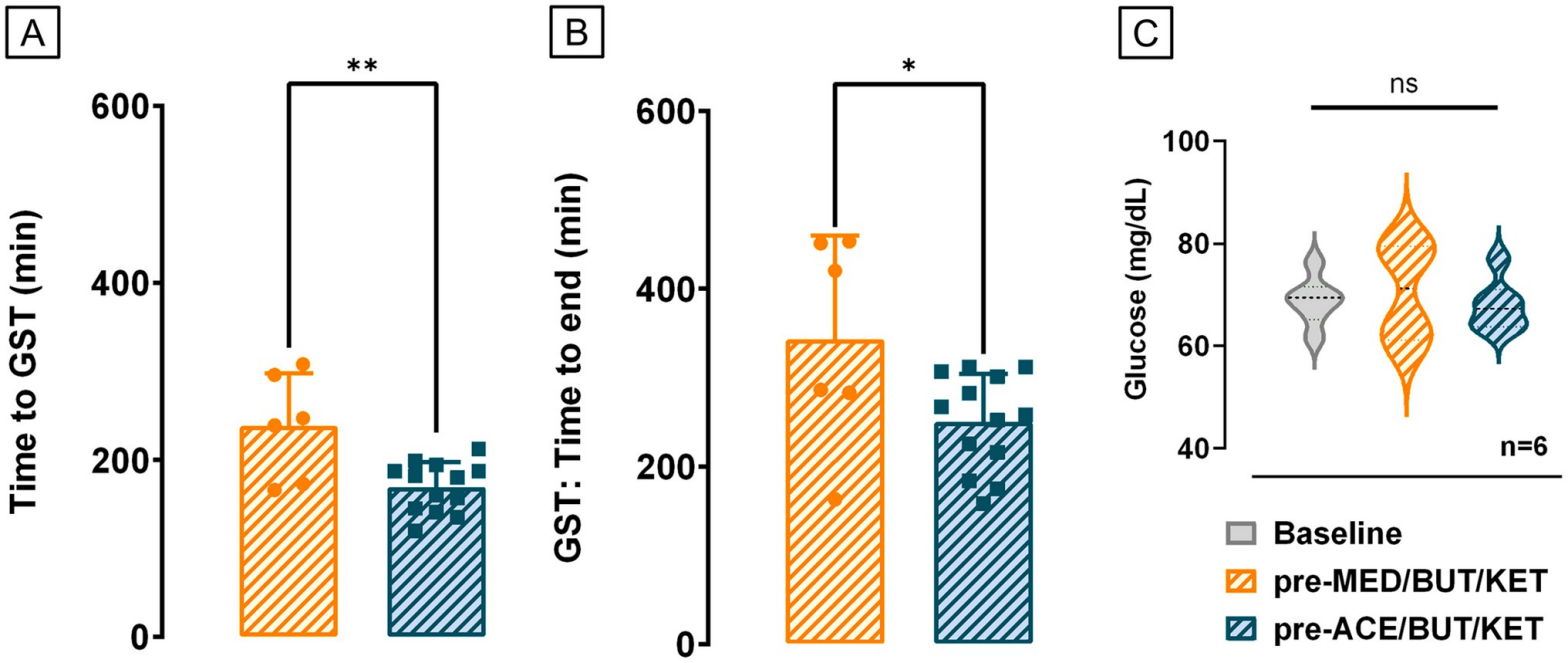
Influence of pre-meds in the duration of anesthesia for GST. (A) Animals pre-medicated with MED-BUT** (n = 6) needed a longer time after induction of anesthesia to start the GST tests compared to ACE-BUT (n = 13), once there was an increase in BGM after MED-BUT administration and the increased BGM lasted longer for MED-BUT compared to the ACE-BUT pre-medicated group. (B) Consequently, the overall time of anesthesia, from the moment of induction with propofol to the moment when Isoflurane was suspended also increased for animals pre-medicated with MED-BUT compared to ACE-BUT (Two tailed paired t-test, *p = 0.028, **p = 0.0028). (C) There was no difference in averaged BGM pre-prandial compared to averaged BGM right before pre-meds, excluding that a previous high BGM delayed GST onset (Repeated measures one-way ANOVA, Bonferroni post-hoc test, *p* = 0.7833). Data is presented as mean (SD).

The effect of the pre-meds on the duration of anaesthesia disregarded procedures when no GST was performed, because in those situations the criteria to define duration was related to how fast the investigators were able to insert or remove the sensors. The time needed to start the GST was dependent on baseline glucose values and was longer when animals were pre-medicated with MED-BUT (n = 6) compared to ACE-BUT (n = 13) (Fig 2A), independently of supplementing with Ketamine. The interval between intubation and start of GST was 238.2 (SD = 59.6) min for MED-BUT and 169.2 (SD = 28.07) min for ACE-BUT (Two tailed paired t- test, *p* = 0.0028). Consequently, the duration of anaesthesia was longer when GST tests were performed under MED-BUT and the addition of Ketamine did not interfere in the overall time to start the GST tests. The overall duration of anaesthesia was measured from the time animals were intubated to the time when Isoflurane was suspended (Fig 2B). The criteria to end anaesthesia was also the return of 3 consecutive values of glucose to the baseline. The time to end anaesthesia during GST tests was 342.7 (SD = 117.4) min for MED-BUT and 249.9 (SD = 54.12) min for ACE-BUT (Two tailed paired t-test, *p* = 0.028). Pre-prandial glucose measurements performed in between days of anaesthesia were compared to glucose right before sedation whenever possible, excluding occasions in which minipigs exhibit an aversive behaviour towards it. There was no difference in averaged glucose pre-prandial in mg/dL compared to averaged glucose right before pre-meds. The pre-prandial glucose was 68.84 (SD = 4.84) mg/dL compared to 70.63 (SD = 9.88) mg/dL before MED-BUT and 67.92 (SD = 5.1) mg/dL before ACE-BUT (n = 6, Repeated measures one-way ANOVA, Bonferroni post-hoc test, *p* = 0.7833), excluding an underling high baseline glucose as the cause of delaying GST onset (Fig 2C).

**Fig 3:**
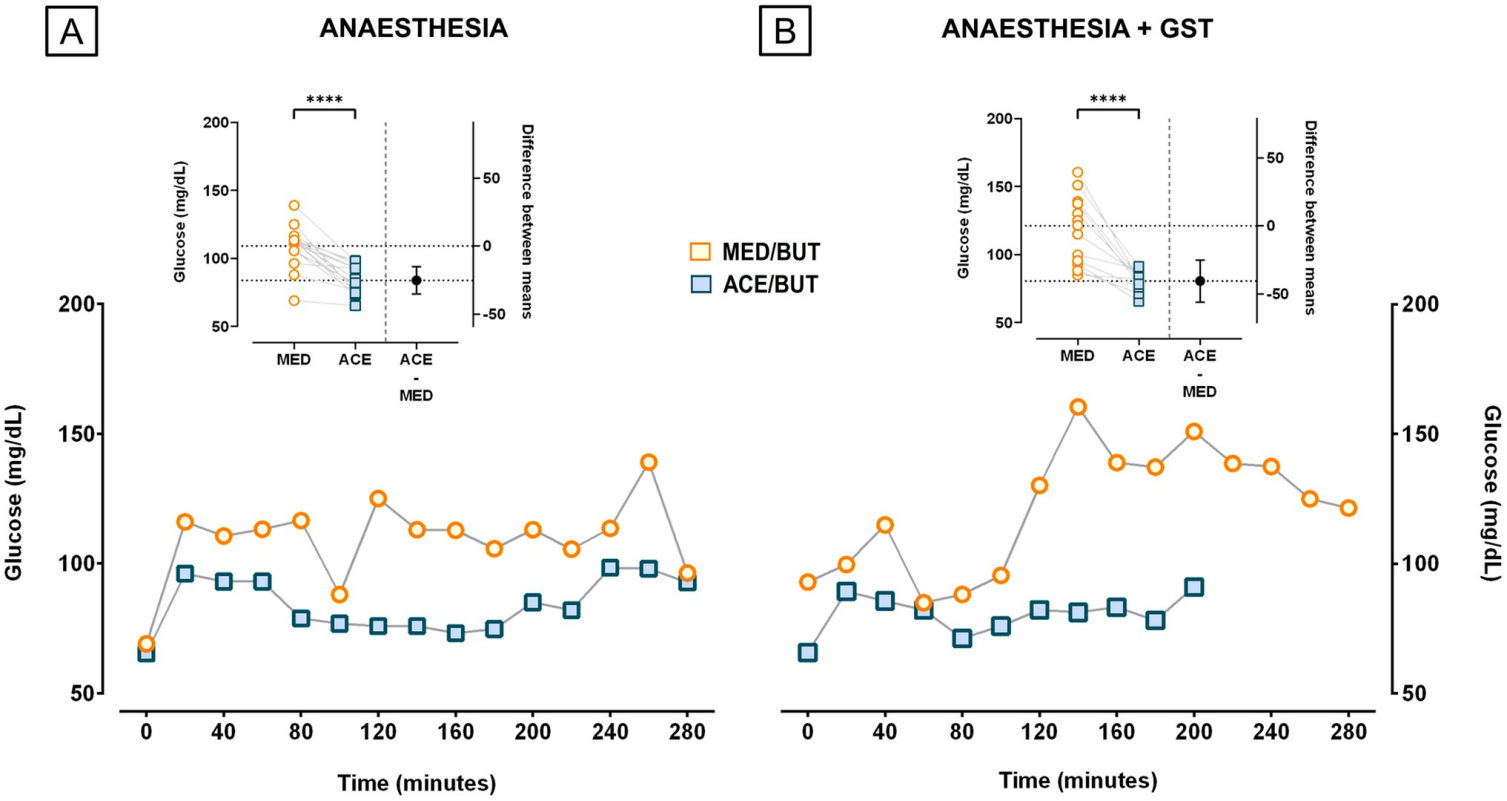
PREMEDs effect in blood glucose. Blood glucose measurements were averaged for all animals and separated into anesthetic procedures for sensors placement and removal and anesthesia to GST tests. (A) A period of 280 minutes is compared between ACE-BUT and MED-BUT with glucose measurements performed every 20 minutes and presented as means. The anesthesia of animals using MED-BUT (n = 9) in the pre-medication presented a higher blood glucose compared to ACE-BUT (n = 9) during almost all analysed times for sensor insertions or removal. (B) When GST’s were performed higher blood glucose values were also observed for MED-BUT (n = 13) in comparison to ACE-BUT (n = 6) group (Two tailed paired t-test, ****p<0.001).

The glucose values were also collected for procedures when anaesthesia was performed to insert or remove glucose sensors, because we were interested in observing the effects of the pre-med on glucose metabolism. When analysing the effects of ACE-BUT (n = 9) or MED-BUT (n = 9) on averaged glucose measures for all animals from time 0 to 280, the glucose was higher for MED-BUT during the anaesthetic procedures for sensors placement or removal (Fig 3A), as well as during anaesthetic procedures for GST tests (Fig 3B) (Two tailed paired t-test, *p*<0.001). It is important to depict that the glucose values displayed in Fig 3B for ACE-BUT (n = 13) or MED-BUT (n = 6) are prior to the peak glucose curves generated by glucagon injection. The hyperglycaemic peaks were analysed separately, but the MED-BUT hyperglycaemic peak in mean (SD) was on average 50.44 (SD = 41.22) higher than the glucose values collected during anaesthesia, compared to a 117.45 (SD = 44.44) increase in glucose after glucagon for the ACE-BUT pre-med group. Additionally, a transient increase in glucose after induction with propofol was observed, lasting no longer than 20 minutes in ACE-BUT pre-medicated animals.

**Fig 4:**
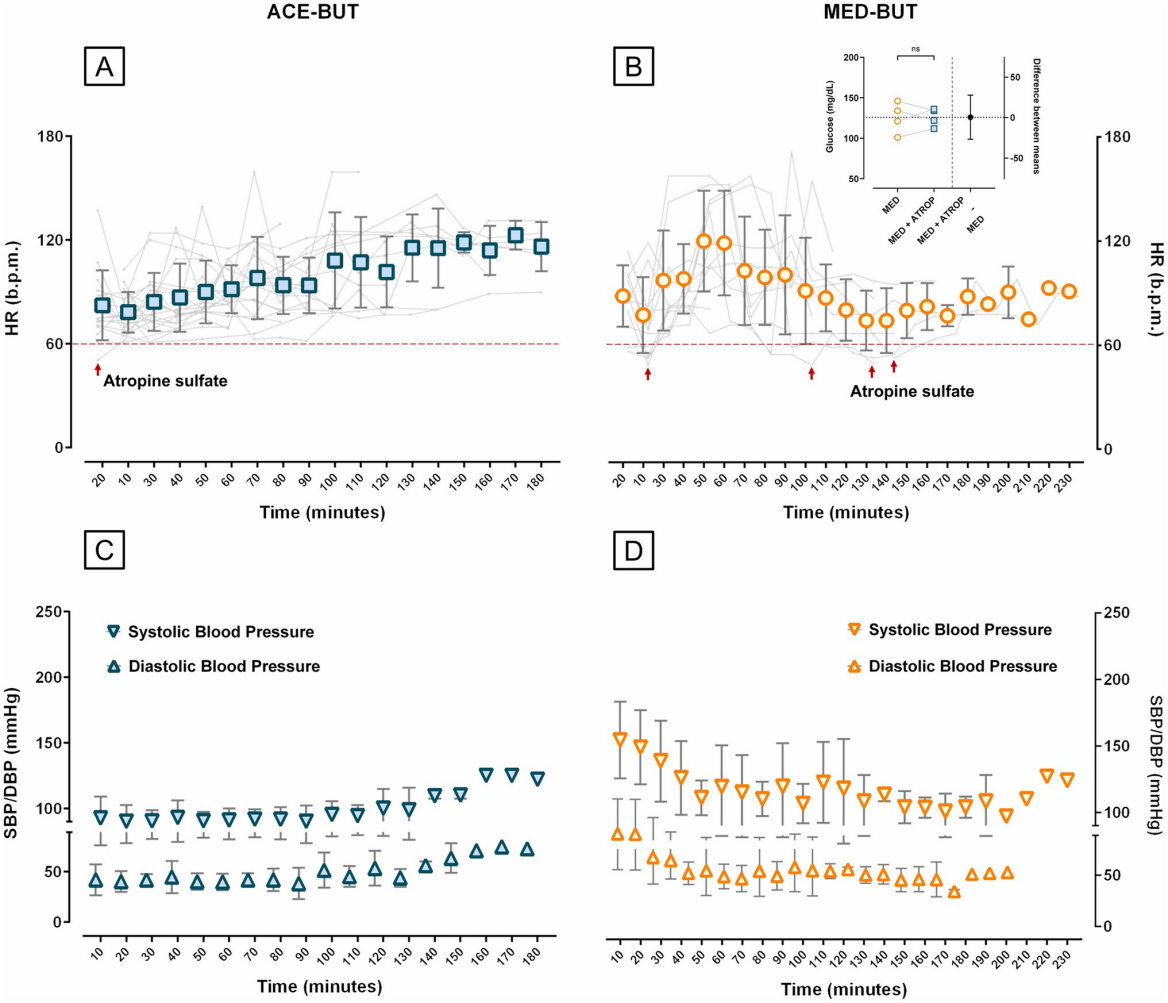
Safety of anaesthesia. The administration of the anti-cholinergic atropine sulfate (red arrows) was necessary in some minipigs to increase HR above the minimum recommended physiological value 60 b.p.m (dashed line). Altogether, 1 minipig anaesthetized with (A) ACE-BUT in comparison to 4 animals that received (B) MED-BUT in the pre-med required atropine. (B, top right panel) Atropine administration did not affect glucose levels in those animals (Two tailed paired t-test, *p* = 0.966). Both systolic and diastolic pressures were maintained in between the physiological range for Aachen Minipigs during the anesthetic procedures using either (C) acepromazine or (D) medetomidine.

The administration of the anti-cholinergic atropine sulfate (0.5-1mg/animal, red arrows) was necessary to counteract bradycardia (< 60 b.p.m, dashed lines) in 1 out of 22 (4.5%) animals anaesthetized with ACE-BUT (Fig 4A) in comparison to 4 out of 15 (27%) minipigs that received MED-BUT (Fig 4B) and considered as an indication of reduced safety of the pre-med. In one of the four minipigs, it was even necessary to repeat atropine during the anesthetic procedure. Note that atropine administration did not affect glucose levels in those animals (inlet in B, top right panel) when comparing glucose measurements collected before and glucose levels measured after atropine administration. The averaged values of glucose withing subjects were 125.5 (SD = 19.26) mg/dL for MED-BUT before atropine and 126 (SD = 11.2) mg/dL after atropine administration (n = 4, Two tailed paired t-test, *p* = 0.966). The blood glucose of the animal in the ACE-BUT group was 72 mg/dL before and 69 mg/dL 30 minutes after atropine administration. Both protocols with acepromazine (Fig 4C) and medetomidine (Fig 4D) maintained systolic and diastolic pressure into the physiological range for minipigs, but fluctuations on pressure were more common among the MED-BUT group.

The improvement caused by the changes in animal handling did not generate a direct objective parameter for comparison, but we observed a general improvement in the relationship with the animals and a reduction in the escaping behaviour of minipigs, since pigs are in general very reactive to human manipulation of any kind. All researchers were approached voluntarily, and the animals demonstrated a clear reduction in the anxiety levels to human contact as the rounds of experiments proceeded throughout the year.

## Discussion

The results indicate that premedication with ACE-BUT provided a weaker sedation when compared to MED-BUT as expected [21], but was superior in safety, providing stable glucose values during the anaesthetic procedures, therefore reducing the overall time of anaesthesia, and optimizing experimental outcomes. Furthermore, the strategies to minimize handling-induced stress showed a reduction in the avoidance behaviour of the minipigs.

### Optimization of the anaesthetic protocol

One of the crucial points for the success of this specific study was to maintain stable glucose levels during the observational period and to do so, in the following paragraphs we review the recommended anaesthetics for swine, taking into consideration the goal of balancing sufficient depth of anaesthesia with minimal interference in the glucose levels. Additionally, we discuss how the anaesthetic planning helped to improve the research outcomes. The discussion aimed to add educational value, not only in improving future diabetes-related studies in minipigs, but also in providing insights on which parameters must be taken into consideration during anaesthetic planning for other scientific purposes.

The mainstream recommendation for pig anaesthesia is to use benzodiazepines (e.g. midazolam, diazepam), or alpha-2 adrenergic agonists (e.g. xylazine, medetomidine, dexmedetomidine) associated with ketamine [19, 21–23]. The combination of midazolam and ketamine is widely described in the literature as safe and effective for sedation and muscle relaxation in swine [19, 24, 25]. However, the effects of benzodiazepines in glucose metabolism seemed controversial, with one study showing it to cause hyperglycemia in humans, mice and rats [13]. Another study in healthy humans, suggested that midazolam in continuous infusion reduces blood glucose levels after 60 min administration [26]. Azaperone, another safe and effective sedative, was shown to increase cortisol concentrations with a single dose administration and elicit a strong stress response in swine. The azaperone induced release of cortisol promoted an increase in plasma glucose concentrations after a single dose administration, which was sustained during the whole observational period [27]. For these reasons, both benzodiazepines and azaperone were avoided in the pre-meds here.

The choice of acepromazine was based on its low interference with the glucose metabolism as previously stated, even if we were aware of the weaker sedative effect in pigs [17, 19] compared to the mainstream recommended pre-meds. The range of recommended acepromazine dosage to be administered in swine varies largely from as low as 0.03 mg/Kg [28] and 0.04 mg/Kg when associated with butorphanol [29] and up to 1.1 mg/Kg in the majority of sources, stating that high dosages should be avoided to prevent hypotension due to peripheral vasodilation and hypothermia [22, 24]. We did not observe hypotension with the dosage range used in this study (maximum 0.8mg/Kg).

While MED-BUT provided a deeper level of sedation, a clear interference with the glucose metabolism was observed as increased glucose levels and longer time needed for the glucose to return to baseline, if possible. The longer time needed for the glucose to return to close to baseline consequently increased the overall duration of anaesthesia. Moreover, MED-BUT resulted in higher fluctuations in HR, increasing the anaesthetic risk for minipigs. The use of atropine in bradycardic minipigs anaesthetized with MED-BUT was undesirable not only for safety reasons, but also because a study in humans has shown that atropine can reduce the insulin release and increase glucose levels [30]. However, the recommended dosage of atropine for swine to revert bradycardia had no effect in the glucose metabolism during our experiments (see inlet Fig 4B, top right panel). The increase in both systolic and diastolic BP, as a compensatory physiological mechanism to low HR, was also observed and attenuated using atropine sulfate. It is well known that both perioperative hypo- and hyper-tension are associated with higher incidences of acute kidney failure, increasing the risk of mortality sixfold [31, 32]. Anaesthetic complications are highly undesirable in any case, but particularly problematic in long- term studies such as ours, where animals remained part of the study for at least 1 year and underwent several anaesthetic procedures.

It is described that propofol can reduce sympathetic nerve activity, resulting in blockade of catecholamine release ultimately not affecting insulin secretion and avoiding acute hyperglycemia [33, 34]. The mechanisms behind the effect of propofol in preventing hyperglycemia are controversial and some authors suggest it could be related to decrease in plasma concentrations of cortisol in stress-free procedures [35]. As mentioned above, we actually noticed a temporary rise in glucose after propofol administration that resolved completely after 20 minutes in the ACE-BUT pre-med group. This could be explained with the very short half-life of propofol after a single bolus injection [36]. In contrast, we observed in the MED-BUT group an increase in glucose levels upon its injection and despite the administration of propofol. This hyperglycemia lasted the entire period of anaesthesia and might be mainly attributed to the effect of medetomidine, even though a propofol-associated effect in combination with medetomidine cannot be excluded completely.

Both sevoflurane and isoflurane can decrease glucose metabolism in the heart and brain, but no alterations in systemic blood glucose level are mentioned. It is also stated that sevoflurane can reduce insulin secretion and increase glucose levels by impairing glucose use [7]. We therefore abandoned sevoflurane during our study and used isoflurane instead. No effect of isoflurane was observed in interfering with glucose levels when looking into the stable glucose levels in the ACE-BUT group during the maintenance of general anaesthesia.

The anaesthetic planning must necessarily include the general aim of avoiding postoperative side effects and pain or distress during induction or recovery, in accordance with EU Directive 2010/63. Cortisol is known to be inhibited by the use of opioids and a human study using Butorphanol demonstrated comparable sedative effects to diazepam and indistinguishable effects when compared to midazolam [37, 38]. Butorphanol was used to add sedation effect, especially in the ACE-BUT group. The dosage was almost identical (0.4–0.5mg/kg), and all animals received butorphanol, therefore it is possible to exclude its effect from the inferences. When a desirable level of sedation was not obtained by either ACE-BUT or MED-BUT the pre-med had to be supplemented with ketamine to allow for vein cannulation and stress-free animal manipulation. We knew that dissociative agents could increase catecholamines, subsequently increasing glucose levels [21], but we did not observe hyperglycemia due to ketamine when comparing readings in the same animal, using the same protocol with or without ketamine. Additionally, the decrease in glucose levels expected after 8h of fasting in pigs [27] was not observed in our study when comparing glucose measured in the days between procedures with glucose right before pre-med administration.

### Animal handling

Besides the choice of anaesthetics, it was also crucial to minimize handling-induced and other sources of stress and cortisol release- stress-induced sympathetic response, i.e. catecholamine release followed by hepatic glucose and lipid elevation [38]. Minimizing animal distress and pain is an ethical obligation and part of the third “R” that stands for refinement. We administered the pre-meds while the minipigs were still in the cages and distracted them by providing a few raisins and brushing the skin concomitantly to the intramuscular injections. Our strategy might not have been as efficient as the zero-stress anaesthesia induction (ZESTRANI) technique, in which sugar cubes are treated with anaesthetics [39], but we no longer experienced an avoidance behaviour, as previously mentioned. Also, intercalating measures with positive reinforcement resulted in volunteer approach of the animals to the researcher’s indicating reduction in fear induced stress.

### Limitations

It is important to mention some limitations we faced during our study. We were unable to measure cortisol levels so we cannot quantify the efficacy of our methods in reducing animal distress and therefore can only describe our subjective observations. Additionally, data referring to the period of recovery from anesthesia is only partially recorded and therefore not included in the results. Based on later literature research, dexmedetomidine would perhaps have been a better option to medetomidine for the maintenance of the glucose levels during the anaesthetic procedures [40, 41] and we suggest it to be tested in similar future studies. The biological replicates in this study were rather small and compensated by a maximum number of sensors per animal, but future studies may be necessary to confirm those findings in a larger population.

## Conclusions

Different animal species and breeds have their own behavioral, anatomical, and physiological characteristics, not to mention individual responses. We have observed that Aachen minipigs indeed have a docile temperament, still some animals were more reactive than others to humans’ manipulation, requiring adjustments in the anaesthetic protocol. It is fundamental that the researcher takes the expected outcomes of a scientific study as well as species-specificities into consideration and adjusts the anaesthetic protocols accordingly. In our specific case, although ACE-BUT has demonstrated a weaker sedative effect and muscular relaxation, the minimal interference with the glucose metabolism and higher safety suggests it to be a better choice as pre-medication for diabetes-related studies in Aachen minipigs.
